# Influence of Combinations of Lipophilic and Phosphate Backbone Modifications on Cellular Uptake of Modified Oligonucleotides

**DOI:** 10.3390/molecules29020452

**Published:** 2024-01-17

**Authors:** Timofey D. Zharkov, Oleg V. Markov, Sergey A. Zhukov, Svetlana N. Khodyreva, Maxim S. Kupryushkin

**Affiliations:** Institute of Chemical Biology and Fundamental Medicine, Siberian Branch of RAS, Lavrentiev Ave. 8, 630090 Novosibirsk, Russia; timazharkov74@gmail.com (T.D.Z.); markov_ov@niboch.nsc.ru (O.V.M.); jsvbsasp@yandex.ru (S.A.Z.); svetakh@niboch.nsc.ru (S.N.K.)

**Keywords:** lipophilic oligonucleotides, phosphate modifications, phosphoryl guanidine, triazinyl phosphoramidate, conjugates, delivery

## Abstract

Numerous types of oligonucleotide modifications have been developed since automated synthesis of DNA/RNA became a common instrument in the creation of synthetic oligonucleotides. Despite the growing number of types of oligonucleotide modifications under development, only a few of them and, moreover, their combinations have been studied widely enough in terms of their influence on the properties of corresponding NA constructions. In the present study, a number of oligonucleotides with combinations of 3′-end lipophilic (a single cholesteryl or a pair of dodecyl residues) and phosphate backbone modifications were synthesized. The influence of the combination of used lipophilic groups with phosphate modifications of various natures and different positions on the efficiency of cell penetration was evaluated. The obtained results indicate that even a couple of phosphate modifications are able to affect a set of oligonucleotide properties in a complex manner and can remarkably change cellular uptake. These data clearly show that the strategy of using different patterns of modification combinations has great potential for the rational design of oligonucleotide structures with desired predefined properties.

## 1. Introduction

At present, modified oligonucleotides are finding new applications in a wide range of scientific and technological fields, including the creation of unique therapeutics and diagnostics [1,2,3,4,5,6,7,8,9,10,11,12]. For example, 18 drugs based on therapeutic nucleic acids (NAs) have already been officially approved for use in the treatment of various human diseases, and all of them contain chemical modifications [1]. Modifications in the oligonucleotide structure provide the enhanced therapeutic efficacy of such drugs by improving some of their most important properties, e.g., efficiency of penetration through the cell membrane [13,14,15,16,17,18]. The absence of a universal solution within the choice of the type of oligonucleotide modification for various tasks has led to the intensive development of new NA modifications. Despite the growing number of types of oligonucleotide modifications under development, only a few of them and, moreover, their combinations have been studied widely enough in terms of their diverse influence on the properties of corresponding NA constructions.

Various phosphate modifications could potentially be used to study the complex impact of backbone modifications and their combinations on oligonucleotide properties. One of the most promising approaches to obtaining NA derivatives is based on altering the protocol of the phosphite-triester oxidation stage during solid-phase synthesis. For instance, phosphorothioates, being the most popular type of phosphate-modified oligonucleotides, can be synthesized in this way [19,20,21,22,23]. This modification could be applied to the preparation of oligonucleotides with even fully modified sugar–phosphate backbones. Phosphorothioate derivatives possess much higher stability to different nucleases [19,20] and improved cellular uptake as well as biodistribution due to their high affinity to proteins compared to unmodified oligonucleotides [21,22]. Considering the chemical approach of phosphorothioate modification synthesis, there is a crucial drawback since only one representative of this class of altered phosphate groups could be obtained using the developed sulfurizing reagents [23], which are not intended to vary functional groups in their structure. Over the past decade, several chemical approaches utilizing alternative oxidation steps to obtain novel structures of modified phosphate groups, which allow one not only to change the nature of the phosphate backbone but also to apply the chemistry of its introduction to varying desirable functional moieties, have been proposed [24,25,26,27,28,29,30,31,32,33]. In particular, we have adopted the method of using electron-deficient azides in the Staudinger reaction during the oxidation step to efficiently obtain various phosphate derivatives [34,35,36,37,38,39]. We have also demonstrated that different representatives of the developed phosphoryl guanidine and triazinyl phosphoramidate modifications can be applied for the synthesis of lipophilic oligonucleotides with enhanced levels of intracellular accumulation [37].

The compatibility of newly developed methods for the incorporation of phosphate modifications [29,38,39] with the standard protocol of automated synthesis, as well as the possibility of combining them with many types of popular phosphoramidite monomers and modifiers, makes them a promising tool for creating oligonucleotides with complex sugar–phosphate backbones, which have recently come into use. For example, the improvement of cell delivery and other beneficial properties of therapeutic oligonucleotides, such as biodistribution, target binding, etc., for antisense [40,41,42,43,44,45,46], siRNA [47,48,49,50,51,52,53] and oligonucleotides with other types of action [54] could be achieved with different combinations of backbone modifications, including altered phosphate groups.

One of the recent examples where the positive effect of a combination of modifications has been shown is the number of studies about the properties of phosphoryl guanidine (PG) oligonucleotides. Despite early published data observing that the introduction of 1,3-dimethylimidazolidin-2-ylidene phosphoramidate (DMI) groups, the most common representative of the PG class, into the structure of the native oligonucleotide did not lead to a detectable increase in the level of cellular uptake [55], it was later shown that the incorporation of such modifications into phosphorothioate oligonucleotide may result in a more than two-fold increase in the level of intracellular accumulation [45]. This example demonstrates that a combination of different modifications may provide synergistic improvements in desired oligonucleotide properties.

In this study, we have chosen an oligonucleotide containing lipophilic dodecyl residues as the initial NA system with a proven significant level of cellular uptake [37] and then introduced two adjacent DMI groups in its backbone structure. Several parameters of the present system were varied in order to evaluate their influence on the intracellular accumulation of corresponding oligonucleotides.

## 2. Results

### 2.1. Design and Synthesis of Lipophilic Phosphate-Modified Oligonucleotides

A 5′-fluorescein-labeled oligonucleotide with a randomized sequence, which was previously used in an intracellular delivery study [37], was chosen as the primary system for evaluation of the impact of DMI groups on the efficiency of intracellular accumulation. This oligonucleotide contains two dodecyl residues introduced through 3′-end internucleotide triazinyl phosphoramidate (TPA) modification (oligonucleotide A, Table 1). The two neighboring DMI modifications were introduced opposite to the TPA part of the sequence (oligonucleotide AX, Table 1). The resulting oligonucleotide has a gapmer-like structure, where phosphate-modified regions of the oligonucleotide flank an unmodified middle part.

In order to evaluate the influence of several structure-related factors on cell penetration, namely the type of lipophilic modification, the nucleotide sequence, the position and number of DMI groups in the oligonucleotide structure, as well as the nature of phosphate backbone modification, an additional set of oligonucleotides was synthesized (Table 1).

A cholesterol residue, being well known and widely used as a delivery-enhancing agent [56,57,58,59,60,61], was selected as the alternative lipophilic group and was introduced in the 3′ end of the oligonucleotide (H) and its DMI-containing analog (HX) using the corresponding commercially available solid support.

To investigate the potential dependence of changes in cell penetration efficiency upon DMI group introduction in the oligonucleotide backbone on the nucleotide sequence, the oligonucleotide pair B and BX containing a lipophilic TPA modification similar to an A and AX pair but with a different sequence was synthesized (Table 1). It should be noted that two different, but both randomized, sequences without any known cellular target have been chosen consciously to avoid their potential biological activity that may interfere with the toxicity or survival of cells and evaluate a distinct effect of oligonucleotide delivery efficiency.

For the determination of the possible effect of the position of the DMI groups on cellular uptake, in addition to BX with the 5′-end localization of these modifications, oligonucleotides also containing two neighboring DMI groups in the middle part of the sequence (BX′, Table 1) and in the 3′-end region directly adjacent to the lipophilic TPA modification (BX″, Table 1) were synthesized. Additionally, to evaluate the impact of varying the number of DMI groups in the structure of oligonucleotide A, a set of derivatives bearing 4, 6, 8, and 10 sequential DMI modifications starting from the 5′-end region was obtained (AX2, AX3, AX4, AX5, Table 1).

Besides changing the lipophilic part of the oligonucleotides, their sequences, the position and number of DMI groups, and the type of introduced phosphate modifications were also varied. Di(pyrrolidine-1-yl)methylene phosphoramidate, another representative of the phosphoryl guanidine modification class [35], was used as a bulkier and more hydrophobic alternative to the DMI group and was introduced into the corresponding oligonucleotide (BY). Methanesulfonyl phosphoramidate modification, being the simplest representative of the alkanesulfonyl phosphoramidate modification class [62], which is nowadays finding new applications [24,27], as well as common phosphorothioate modification, was also used to alter the oligonucleotide backbone (BM and BS, respectively, Table 1). It should be noted that all types of considered PN-containing modifications (TPA, DMI, di(pyrrolidine-1-yl)methylene phosphoramidate, and methanesulfonyl phosphoramidate) were introduced into the oligonucleotide backbone using a single approach based on the Staudinger reaction involving electron-deficient azides during the alternative oxidation step. Thus, the Staudinger reaction in solid-phase phosphoramidite DNA/RNA synthesis could be considered a single platform for creating a wide range of phosphate-modified oligonucleotides.

HPLC was used for the purification of the synthesized oligonucleotides. The molecular masses of the oligonucleotides were confirmed by ESI mass spectrometry. The HPLC profiles of the reaction mixtures (Figure S1), purified solutions (Figure S2) of oligonucleotides, data of the mass spectrometry analysis (Figure S3), and melting temperatures of the duplexes (Table S1) are presented in the “Supplementary Materials”. To sum up, it was shown that the applied methods for the synthesis of lipophilic phosphate-modified oligonucleotides possess high efficiency, allowing different types of phosphate modifications to be combined.

### 2.2. Dynamic Light Scattering (DLS) Analysis of Supramolecular Complexes of Lipophilic Phosphate-Modified Oligonucleotides

**Figure 1:**
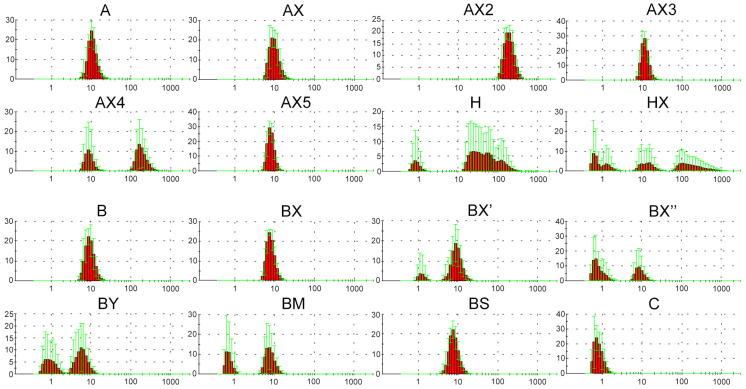
Number graphs (red histograms) of the DLS experiment for the lipophilic oligonucleotides used in the current study after 2 h incubation in serum-free DMEM medium, 10 measurements. X axis is diameter size, nm; Y axis is number, percent; green lines are error bars, SD.

Previously, the possibility of forming various supramolecular complexes with dodecyl-containing oligonucleotides was demonstrated [63]. Taking this into consideration, we decided to characterize the solution of the obtained oligonucleotides using DLS measurements under conditions equal to those of transfection [37] as the most relevant for the identification and correct interpretation of the influence of various parameters of the oligonucleotide system on cellular uptake. It was shown that in selected conditions (serum-free DMEM medium, 2 h, 10 measurements), oligonucleotide A containing a lipophilic TPA modification can form stable supramolecular complexes. The obtained data reliably differ from those for DMEM or DMEM with control unmodified oligonucleotide (Figure S4). All synthesized lipophilic oligonucleotides were also analyzed using the DLS method in order to evaluate the influence of introduced phosphate modifications on the possibility of forming supramolecular complexes over the same experimental conditions (Figure 1).

The results of the DLS experiment showed that oligonucleotides A and B both form stable supramolecular complexes with hydrodynamic diameters of about 10 nm. Moreover, the introduction of DMI groups in the 5′-end region (AX and BX) did not lead to any significant changes in complex formation (Figure 1). The oligonucleotides H and HX bearing a cholesterol moiety instead of a dodecyl-containing TPA modification were shown to form a number of irregular or unstable complexes with a wide range of hydrodynamic diameters, independent of the presence of DMI modifications. Consecutive changes in the position of a pair of neighboring DMI groups from the 5′- to the 3′-end region of the oligonucleotide structure (BX, BX′, and BX″, respectively) led to a sequential decrease in the efficiency of complex formation. However, a sequential increase in the number of introduced DMI groups in the same direction (5′ → 3′-end region) did not result in any regularities within complex formation. Thus, oligonucleotide AX2 containing four DMI modifications forms stable complexes with hydrodynamic diameters of more than 100 nm, and at the same time, oligonucleotides AX3 (six DMI groups) and AX5 (ten DMI groups) assemble into complexes similar to those formed by oligonucleotide AX (two DMI groups). Oligonucleotide AX4 (eight DMI groups) is capable of forming both types of complexes with hydrodynamic diameters of 10 and more than 100 nm. Interestingly, the nature of even two introduced phosphate modifications (oligonucleotides BX, BY, BM, and BS) also can influence the formation of supramolecular structures. Thus, the introduction of DMI (BX) or phosphorothioate (BS) modifications into the oligonucleotide backbone did not lead to significant changes in complex formation, whereas the incorporation of a bulkier representative of the phosphoryl guanidine class (BY) as well as methanesulfonyl phosphoramidate modifications (BM) resulted in the formation of less stable complexes. All of the abovementioned points out the unobvious manner of the influence of modifications and their combinations on the properties of the resulting oligonucleotide structures.

### 2.3. Study of the Efficiency of Intracellular Accumulation of Phosphate-Modified Oligonucleotides by Flow Cytometry and Confocal Microscopy

**Figure 2:**
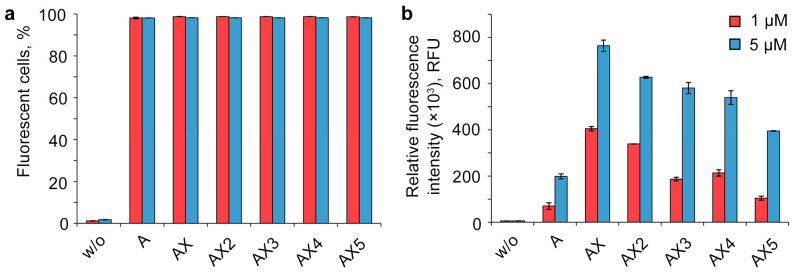
Effects of the number of DMI modifications on the cellular uptake of FAM-labeled lipophilic phosphate-modified oligonucleotides. Analysis was performed in HEK293T cells 4 h post-transfection. w/o—untreated cells. Percentage of fluorescent cells (**a**) and relative fluorescence intensity (**b**) were measured using flow cytometry. Data are presented as mean ± SD.

For quantitative assessment of the efficiency of intracellular accumulation of oligonucleotides with different numbers of DMI groups, transfection was carried out for 4 h in a serum-free DMEM medium with a 1 or 5 μM concentration of oligonucleotides in the HEK293T cell line, which has been proved to be a convenient model to study the cellular uptake of NA derivatives [34,36,37,64,65]. The transfected cells were analyzed using flow cytometry (Figure 2). To evaluate the transfection efficiency, two parameters were used: the percentage of fluorescent cells (Figure 2a) and the relative fluorescence intensity (Figure 2b). The untreated cells served as a negative control. All of the modified oligonucleotides accumulated in almost 100% of the cells under the experimental conditions, and hence their mean fluorescent intensity can be compared directly.

One of the most unexpected results of the experiment is the manner of cellular uptake of oligonucleotides bearing several DMI groups. The initial introduction of DMI groups in the 5′-end region (Figure 2, oligonucleotides A and AX) led to a significant rise in the intracellular accumulation level. However, the sequentially increasing number of DMI groups resulted in a gradually decreasing uptake efficiency of the corresponding multiple modified oligonucleotide derivatives, despite the growth of the overall hydrophobicity (Figure 2, AX–AX5).

Taking into account significant differences in the sugar–phosphate backbone of oligonucleotides with various numbers of DMI groups, as well as their different cellular uptake levels, we decided to compare the kinetics of intracellular accumulation of oligonucleotides with two (AX) and ten (AX5) DMI groups.

**Figure 3:**
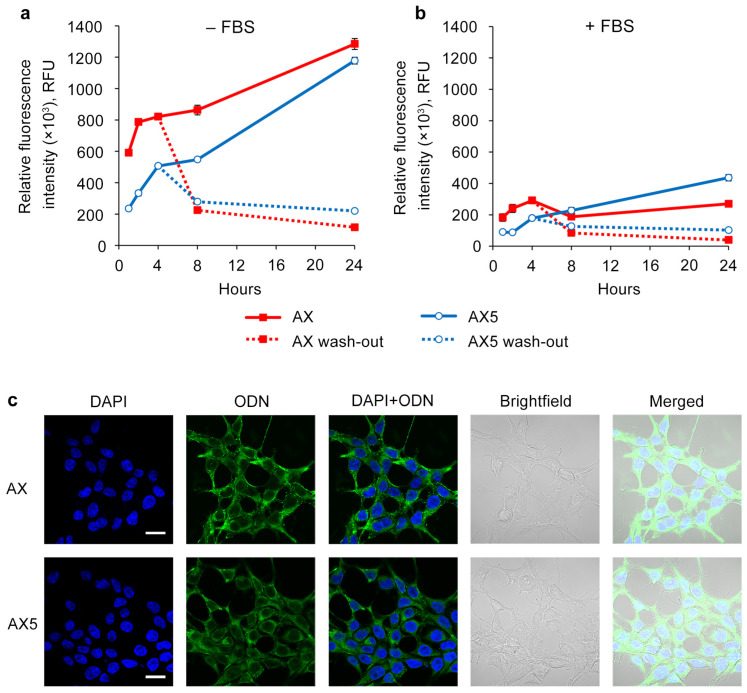
Kinetics of intracellular accumulation of AX and AX5 oligonucleotides (5 µM) in HEK293T cells. Transfection was performed in serum-free (**a**) or serum-containing (**b**) medium. After 4 h of transfection, cells were washed with PBS and refreshed with culture medium in a wash-out regimen. The relative fluorescence intensity was measured by flow cytometry. Data are presented as mean ± SD. (**c**) Intracellular distribution of AX and AX5 oligonucleotides (5 µM) in HEK293T cells 4 h post-transfection. ODN—FAM-labeled oligodeoxynucleotides (green channel). Analysis was performed with a Zeiss LSM710 confocal microscope using a Plan-Apochromat 63×/1.40 Oil DIC M27 objective. Nuclei were stained with DAPI (blue channel). Scale bars 20 µm.

Transfection was carried out for 1, 2, 4, 8, and 24 h in a serum-free medium or complete DMEM using a 5 μM concentration of oligonucleotides on the HEK293T cell line (Figure 3a). It was shown that in early time points, e.g., prior to 8 h, oligonucleotide AX accumulates more efficiently compared to AX5 in both types of medium. However, by 24 h, the intracellular accumulation levels of AX and AX5 were nearly equal in serum-free transfection conditions, and in the presence of serum, AX5 demonstrated an even higher level of accumulation.

To investigate the ability of the studied oligonucleotides to stay inside the cells, it was decided to evaluate the intracellular oligonucleotide quantity level over time after 4 h of transfection with subsequent removal of the oligonucleotide solutions and medium changing (Figure 3b). It was shown that at the point of 4 h after the end of transfection, derivatives AX and AX5 demonstrated nearly equal levels, and at the point of 20 h, AX5 possessed a higher level of intracellular presence in both types of medium. It can be concluded that AX5 accumulates more slowly but persists longer than AX once inside the cells.

The presented results of confocal microscopy indicate that the intracellular distribution for oligonucleotide AX did not significantly differ from that previously shown for the initial oligonucleotide system (A) [37]: FAM-labeled oligonucleotides were distributed throughout the cytoplasm in both disassembled and aggregated forms without attaching to the inner side of the cell membrane; no co-localization with nuclei was revealed (Figure 3c, AX). The distribution of oligonucleotide AX5 was similar to that of AX, but with the prevalence of the disassembled form (Figure 3c, AX5), despite both of them being capable of forming supramolecular complexes with the same hydrodynamic diameter under the transfection conditions (Figure 1, AX and AX5).

**Figure 4:**
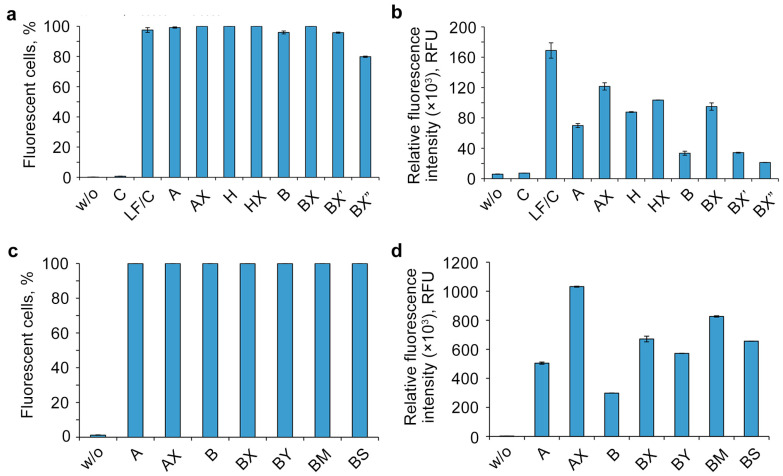
Effects of oligonucleotide sequence, hydrophobic groups, and type and location of phosphate modification on the cellular uptake of lipophilic phosphate-modified oligonucleotides. The cellular accumulation of FAM-labeled lipophilic oligonucleotides (5 µM) in HEK293T cells was measured by flow cytometry 4 h post-transfection. w/o—untreated cells; LF—Lipofectamine 2000. (**a**,**c**)—percentage of fluorescent cells; (**b**,**d**)—relative fluorescence intensity. Data are presented as MEAN ± SD.

The efficiency of intracellular accumulation of other lipophilic derivatives was analyzed using flow cytometry in a serum-free DMEM medium for 4 h of incubation and a 5 μM concentration of oligonucleotides. Unmodified oligonucleotide (C) was analyzed both individually as a negative control and with the commercially available delivery agent Lipofectamine 2000 (LF) as a positive control (Figure 4).

The analysis of the flow cytometry data showed that the other studied factors besides the number of DMI groups (Figure 4) are able to remarkably influence the accumulation efficiency of the investigated lipophilic phosphate-modified oligonucleotides. Dodecyl- and cholesterol-containing conjugates (Figure 4b, oligonucleotides A, B, and H) have an increased cellular uptake, as shown previously [34,36,37,58]. The contribution of not only the different nature of the lipophilic part (A vs. H) but also of the nucleotide sequence (A vs. B) to the delivery efficiency was observed with fluorescence signal differences up to several times (Figure 4b).

One of the important findings is a remarkable increase in intracellular accumulation in all studied oligonucleotides upon introduction of phosphate modifications in the 5′-end region, regardless of the nucleotide sequence (A/AX vs. B/BX), the lipophilic part of the conjugate (A/AX and H/HX), or the nature of the phosphate modification (B/BX/BY/BM/BS) (Figure 4b,d). Despite the relatively small contribution of phosphate modifications to the total hydrophobicity of oligonucleotides containing the 3′-end region lipophilic TPA modification, adding just two groups was shown to significantly improve their delivery efficiency. It should be noted that the contrast in the observed effect diminishes with the introduction of DMI groups in a cholesterol-containing oligonucleotide (Figure 4b, A/AX vs. H/HX,). Another important finding is that the efficiency of delivery is also strongly influenced by the relative position of DMI modifications and the lipophilic TPA group in the oligonucleotide sequence (oligonucleotides BX, BX′, and BX″): as these groups gradually converge, the level of intracellular accumulation decreases (Figure 4b).

**Figure 5:**
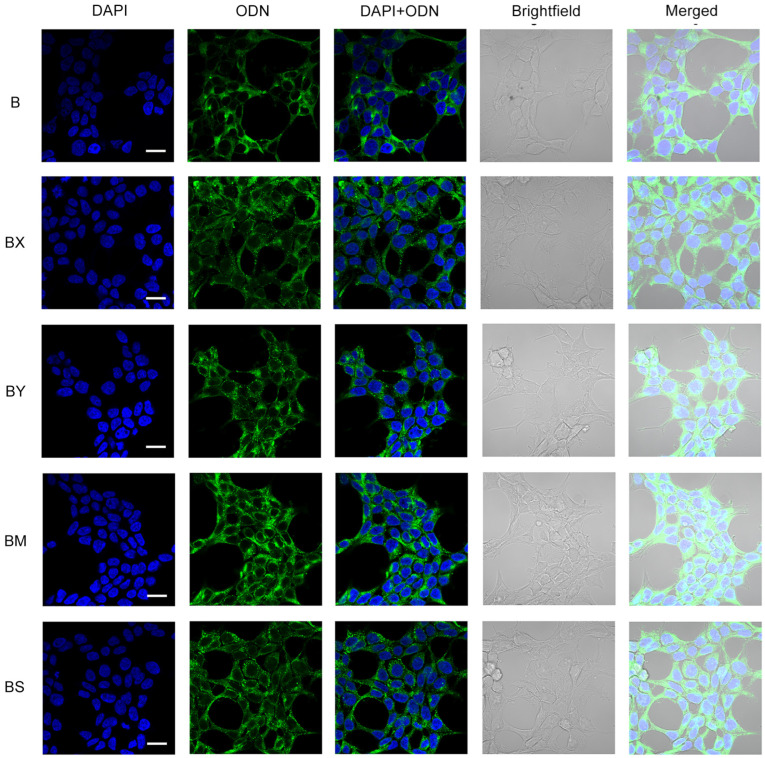
Intracellular distribution of lipophilic phosphate-modified oligonucleotides (5 µM) in HEK293T cells 4 h post-transfection. ODN—FAM-labeled oligodeoxynucleotides (green channel). Analysis was performed on a Zeiss LSM710 confocal microscope using a Plan-Apochromat 63×/1.40 Oil DIC M27 objective. Cell nuclei were stained with DAPI (blue channel). Scale bars 20 µm.

In addition to cellular uptake studies using flow cytometry, the resulting lipophilic derivatives containing a TPA group and different phosphate backbone modifications (B/BX/BS/BM/BY) were analyzed with confocal microscopy in order to reliably determine their accumulation within HEK293T cells. Transfection was carried out under incubation conditions for 4 h in a serum-free medium (DMEM) and an oligonucleotide concentration of 5 µM (Figure 5).

Confocal microscopy demonstrated that the intracellular distribution of the lipophilic B oligonucleotide was characterized by a mostly diffuse disposition in the cytoplasm with a small amount of aggregated forms and without co-localization with nuclei (Figure 5, B). The introduction of two phosphate modifications of various types (BX, BY, BM, BS) at the 5′ end of the oligonucleotide resulted in a significant increase in the number of aggregated oligonucleotide forms. Among the studied lipophilic phosphate-modified oligonucleotides, the BM oligonucleotide was characterized by more efficient accumulation but in a more co-localized aggregated form compared to other studied derivatives, including AX5 (Figure 5, BM; Figure 3c, AX5).

## 3. Discussion

The use of different combinations of modifications in the design of synthetic oligonucleotide structures has become a popular trend in recent years. Modern oligonucleotide therapeutic candidates contain not only multiple modifications of the same type but also various combinations of modifications. The design principles of such complex modified oligonucleotides are becoming the basis for the creation of a new generation of antisense and siRNA agents.

Phosphate modifications represent a versatile tool for creating modified oligonucleotides. One of the most convenient approaches for obtaining phosphate-modified oligonucleotides is based on the alternative oxidation step in the protocol of the standard solid-phase phosphoramidite method of synthesis. For example, phosphorothioate modifications, which can be obtained by this approach, are widely used in creating various NA constructions, including complex oligonucleotide structures with combinations of different modifications [1,2,4,7,45]. Previously, we developed another way of carrying out the alternative oxidation step, based on the application of the Staudinger reaction involving electron-deficient azides. It allows one to introduce a wide range of representatives of different classes of phosphate modifications into the obtained sequences without significant changes in the protocols of oligonucleotide synthesis. Moreover, the approach is suitable for introducing various combinations of phosphate modifications using a single platform to obtain complex modified structures, which was demonstrated in the current study.

In the present work, dodecyl-containing triazinyl phosphoramidate was used as a lipophilic modification providing enhanced cellular accumulation and was combined with other types of phosphate modifications in a backbone of synthesized oligonucleotides by applying the developed single platform based on the Staudinger reaction. One of the important findings was a two-fold increase in cellular uptake upon the introduction of two DMI groups at the 5′-end region of the oligonucleotide structure (A to AX), despite only a slight increase in overall hydrophobicity. Subsequently, additional parameters of the studied system were examined for their influence on intracellular accumulation.

The introduction of DMI groups was found to have a positive effect regardless of the oligonucleotide sequence (A to AX and B to BX). At the same time, replacing the lipophilic part with a cholesterol residue led to the preservation of the positive effect upon the introduction of the DMI group; however, it became less pronounced (A to AX and H to HX). Changing the position of DMI groups in the oligonucleotide structure had a major impact on the studied properties: a gradual convergence of the DMI and lipophilic moiety positions led to decreased cellular uptake as well as lowered supramolecular complex stability (BX, BX′, and BX″).

The influence of the nature and number of phosphate modifications on the properties of NA constructions turned out to be the least predictable. For example, oligonucleotide BY was shown to have a lower transfection efficiency than BX, even though the phosphoryl guanidine modification in the BY structure possesses more hydrophobicity than BX. The gradual increase in the number of introduced DMI groups in dodecyl-containing oligonucleotides (AX to AX5) has also resulted in lowered levels of cellular uptake, despite the growth of overall hydrophobicity, but an enhanced retention rate of oligonucleotides inside the cells. The possibility of forming supramolecular complexes also differs for oligonucleotides BX and BY in a non-obvious manner, as well as for derivatives with the other types of altered phosphate (BM, BS) or different numbers of introduced DMI groups (AX to AX5).

It is worth noting that all studied phosphate-modified oligonucleotides represent the mixtures of all possible diastereomers. In works [43,44,53,54], it was clearly shown that the use of stereopure instead of stereorandom oligonucleotides can enhance the positive effect of introducing different combinations of phosphate modifications even more. Therefore, not only the chemistry but also the stereochemistry of the backbone structure should be considered as an important factor that influences the properties of designed oligonucleotides.

The obtained results demonstrate the complex and unobvious influence of phosphate modifications in the oligonucleotide backbone on the properties of the synthesized lipophilic derivatives. Revealing such effects and their systematization is important from the viewpoint of rational design of oligonucleotides containing combinations of modifications and indicates the relevance of the search for various effective modification patterns.

## 4. Materials and Methods

### 4.1. Oligonucleotide Synthesis

The standard phosphoramidite solid-phase synthesis of all modified and unmodified oligonucleotides containing phosphodiester linkages was carried out on an ASM-800 DNA/RNA synthesizer (Biosset, Novosibirsk, Russia). Oligonucleotides were synthesized at the 0.4 µmol scale, using standard commercial 2-cyanoethyl deoxynucleoside phosphoramidites and CPG solid supports (Glen Research, San Diego, CA, USA).

The insertion of triazinyl phosphoramidate (TPA) modification bearing two dodecyl residues in appropriate oligonucleotide structures using a triazine modifier during the modified protocol of the oxidation step was performed as described in [34].

The insertion of DMI modification in appropriate oligonucleotide structures during the modified oxidation step was performed using commercial 2-azido-1,3-dimethylimidazolidinium hexafluorophosphate (TCI, Tokyo, Japan) to obtain the 1,3-dimethylimidazolidin-2-iylidene phosphoramidate structure (DMI) as described in [38].

The synthesis of a pyrrolidine phosphoryl guanidine modifier (azidodipyrrolidinocarbenium hexafluorophosphate) was performed as described in [35].

The synthesis of methanesulfonyl azide was performed as described in [66].

For the introduction of methanesulfonyl phosphoramidate (oligonucleotide BM) and pyrrolidine-containing phosphoryl guanidine (oligonucleotide BY) modifications, the following procedure was performed. The corresponding monomer was coupled according to a special protocol including a standard coupling step followed by an oxidation step. At this step, the Staudinger reaction was performed. A solution of the corresponding azide in acetonitrile was pumped through a column portion-wise. Next, the capping and deblocking stages were performed, and all the following procedures were performed according to the standard protocol of automatic solid-phase phosphoramidite synthesis.

Conditions of the Staudinger reaction were as follows: To obtain the methanesulfonyl phosphoramidate group (oligonucleotide BM), a 0.25 M solution of methanesulfonyl azide in acetonitrile was used, and the reaction proceeded for 1 h; to obtain the pyrrolidine-containing phosphoryl guanidine group (oligonucleotide BY), a 0.25 M solution of azidodipyrrolidinocarbenium hexafluorophosphate in acetonitrile was used, and the reaction proceeded for 1 h.

The insertion of a phosphorothioate modification (PS) in appropriate oligonucleotide structures was performed using 3-((dimethylaminomethylidene)amino)-3H-1,2,4-dithiazole-3-thione (DDTT, Sulfurizing Reagent II) from Glen Research (San Diego, CA, USA) according to the manufacturer’s protocol.

The synthesis of cholesterol-containing oligonucleotides was performed using modified CPG (Primetech, Minsk, Belarus) according to the manufacturer’s protocol.

The introduction of 6-carboxyfluoresceine (FAM) was performed using the corresponding phosphoramidite (Lumiprobe, Moscow, Russia) as described in [37].

### 4.2. Oligonucleotide Purification and Identification

Analytical RP HPLC was performed using a Millichrom A02 system equipped with a ProntoSIL-120-5-C18 column 2 × 75 mm (Econova, Novosibirsk, Russia) in a linear gradient of acetonitrile 0–50% or 0–90% in 20 mM triethylammonium acetate, pH 7.0, at a flow rate of 200 µL/min, and with detection at 260, 280, and 300 nm wavelengths. Analysis of the derivatives bearing cholesterol moieties (H, HX) was performed through reverse-phase HPLC analysis on an Agilent 1200 HPLC system using a Symmetry300 C4 5 μm column 4.6 × 150 mm (Waters, Milford, MA, USA) under the same conditions.

For oligonucleotide purification, RP HPLC was used on an Agilent 1200 HPLC system equipped with a Zorbax SB-C18 5 μm column 4.6 × 150 mm (Santa Clara, CA, USA) or a Symmetry300 C4 5 μm column 4.6 × 150 mm (Waters, MA, USA) in a linear gradient of acetonitrile 0–50% or 0–90% in 20 mM triethylammonium acetate, pH 7.0, at a flow rate of 1.5 mL/min, and with detection at 260, 280, 300, and 500 nm wavelengths. Desired fractions were collected and concentrated, and the oligonucleotides were precipitated with 2% LiClO_4_ in acetone. The precipitates were separated via centrifugation, washed with acetone, dried in air, and dissolved in deionized water.

The molecular weights of oligonucleotides were determined via ESI-MS on an Agilent G6410A mass spectrometer (Santa Clara, CA, USA) in negative ion mode. The oligonucleotide samples were dissolved in 20 mM triethylammonium acetate in 60% aq. acetonitrile at a concentration of 0.1 mM. The analysis was performed in 80% aq. acetonitrile with a flow rate of 0.1 mL/min. Standard settings of the mass spectrometer were used. Molecular masses were calculated from the experimental m/z values obtained for each sample.

### 4.3. Characterization of Modified Oligonucleotides by DLS

The size distributions of supramolecular complexes of modified oligonucleotides were determined with a dynamic light scattering technique using a Zetasizer Nano-ZS (Malvern Panalytical Ltd., Malvern, UK) at 25 °C. The oligonucleotides were dissolved in DMEM medium (Sigma Aldrich, St. Louis, MO, USA) to a 5 μM concentration and then analysis of the size was conducted for 2 h with a period of 10 min between measurements.

### 4.4. Analysis of Intracellular Accumulation of Oligonucleotides

HEK293T cells were pre-seeded at a density of 75,000–150,000 cells/well in DMEM medium (Sigma-Aldrich, St. Louis, MO, USA) containing 10% fetal bovine serum (HyClone, GE Healthcare, Chicago, IL, USA) and 1% antibiotics (MP Biomedicals, Santa Ana, CA, USA) (hereafter, complete medium) in 24-well plates. The cells were then incubated for 18 h at 37 °C in a humidified atmosphere with 5% CO_2_ (hereafter, standard conditions) to allow for adhesion. The serum- and antibiotic-free DMEM supplemented with 1 or 5 µM of the lipophilic phosphate-modified oligonucleotides replaced the culture medium. The cells were then transfected for 4 h under standard conditions. Afterward, the cells were washed with PBS, trypsinized, quenched with complete DMEM medium, pelleted, washed in PBS, and fixed in 2% formaldehyde in PBS. Cellular uptake of oligonucleotides was assessed using a NovoCyte flow cytometer (ACEA Biosciences, Santa Clara, CA, USA). The NovoExpress software v. 1.1.0 (ACEA Biosciences, Santa Clara, CA, USA) was used to analyze the flow cytometry data. The experiments were conducted in triplicate for statistical analysis.

In the kinetics experiment, HEK293T cells were transfected with 5 µM of oligonucleotides in the absence (−FBS) or the presence (+FBS) of 10% FBS in the culture medium. Analysis of cell uptake of oligonucleotides was performed 1, 2, 4, 8, and 24 h after the start of transfection. In the wash-out regimen, transfection of HEK293T cells with oligonucleotides was performed in −FBS or +FBS conditions for 4 h followed by refreshing with complete DMEM medium and incubated under standard conditions until analysis (8 and 24 h after start of transfection).

### 4.5. Confocal Microscopy

HEK293T cells were pre-seeded on glass coverslips (Marienfeld, Lauda-Königshofen, Germany) in 24-well plates (150,000 cells/well) in complete DMEM medium and incubated for 18 h under standard conditions to allow for adhesion. The cells attached to the coverslips were incubated in serum- and antibiotic-free DMEM medium in the presence of 5 µM of the respective lipophilic phosphate-modified oligonucleotides for 4 h under standard conditions. After transfection, the coverslips with cells were washed twice with PBS. Then, the cells were fixed with 4% formaldehyde in PBS for 15 min at 37 °C and washed twice with PBS. The cells were stained with DAPI solution (1:100 in PBS, Thermo Fisher Scientific, Waltham, MA, USA) at room temperature in the dark for 10 min. After staining the nuclei, the cells were washed twice with PBS and placed on glass slides in Fluoromount-G^®^ (SouthernBiotech, Birmingham, AL, USA). The mounted samples were left to cure for 24 h at room temperature in the absence of light. The intracellular localization of lipophilic phosphate-modified oligonucleotides was evaluated using a confocal laser scanning microscope LSM710 (Zeiss, Oberkochen, Germany) equipped with a Plan-Apochromat 63×/1.40 Oil DIC M27 objective. The confocal microscopic images were captured using ZEN Black Edition software v. 8.1 (Zeiss, Oberkochen, Germany).

### 4.6. Statistical Analysis

All statistical analyses were performed using GraphPad Prism v. 8.0.1 software (GraphPad Software Inc., San Diego, CA, USA). Differences among the means were processed using a one-way ANOVA followed by Tukey’s post hoc test (a *p*-value less than 0.05 was considered statistically significant).

## Figures and Tables

**Table 1:** A list of modified oligonucleotides used in this study. [FAM]—5′-terminal fluoresceine moiety; [Chol]—3′-terminal cholesterol moiety. Internucleotide phosphate modifications: *—dodecyl-containing triazinyl phosphoramidate (TPA); ^X^—1,3-dimethylimidazolidin-2-iylidene phosphoramidate (DMI); ^Y^—di(pyrrolidine-1-yl)methylene phosphoramidate; ^M^—methanesulfonyl phosphoramidate; ^S^—phosphorothioate. Retention time (Rt) of oligonucleotides was defined through RP HPLC with the use of C18 column or C4 column (^a^). Yields of oligonucleotides determined from areas in HPLC profiles, n.d.—not determined.

Code	Sequence 5′→3′	Yields, %	Rt (min)	MS (Calc/Found)
A	5′-[FAM]CTGACTATGAAGTAT*T-3′	70	9.6	5877.5/5876.5
AX	5′-[FAM]**^X^**C**^X^**TGACTATGAAGTAT*T-3′	80	9.7	6067.8/6067.6
AX2	5′-[FAM]**^X^**C**^X^**T**^X^**G**^X^**ACTATGAAGTAT*T-3′	n.d.	11.5	6254.8/6253.8
AX3	5′-[FAM]**^X^**C**^X^**T**^X^**G**^X^**A**^X^**C**^X^**TATGAAGTAT*T-3′	n.d.	11.7	6444.9/6446.0
AX4	5′-[FAM]**^X^**C**^X^**T**^X^**G**^X^**A**^X^**C**^X^**T**^X^**A**^X^**TGAAGTAT*T-3′	n.d.	11.8	6635.1/6637.0
AX5	5′-[FAM]**^X^**C**^X^**T**^X^**G**^X^**A**^X^**C**^X^**T**^X^**A**^X^**T**^X^**G**^X^**AAGTAT*T-3′	n.d.	12.1	6825.3/6827.0
H	5′-[FAM]CTGACTATGAAGTATT[Chol]-3′	95	15.0 ^a^	6188.7/6189.0
HX	5′-[FAM]**^X^**C**^X^**TGACTATGAAGTATT[Chol]-3′	99	20.0 ^a^	6375.6/6376.5
B	5′-[FAM]AGTCTCGACTTGCTAT*T-3′	70	9.4	6130.4/6132.0
BX	5′-[FAM]**^X^**A**^X^**GTCTCGACTTGCTAT*T-3′	50	9.6	6320.6/6321.6
BX′	5′-[FAM]AGTCTCG**^X^**A**^X^**CTTGCTAT*T-3′	30	9.6	6320.6/6322.0
BX″	5′-[FAM]AGTCTCGACTTGCT**^X^**A**^X^**T*T-3′	40	9.9	6320.6/6322.0
BY	5′-[FAM]**^Y^**A**^Y^**GTCTCGACTTGCTAT*T-3′	80	9.8	6428.7/6430.0
BM	5′-[FAM]**^M^**A**^M^**GTCTCGACTTGCTAT*T-3′	60	9.7	6284.4/6286.0
BS	5′-[FAM]**^S^**A**^S^**GTCTCGACTTGCTAT*T-3′	60	9.7	6162.4/6163.5
C	5′-[FAM]AGTCTCGACTTGCTATT-3′	90	5.3	5686.0/n.d.
	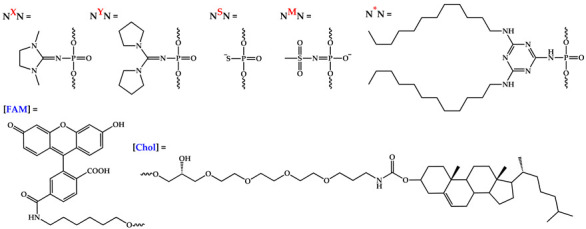			

## Data Availability

All data can be easily obtained and linked in the respective sections.

## Supplementary Materials

The following supporting information can be downloaded at: https://www.mdpi.com/article/10.3390/molecules29020452/s1, Figure S1: Analytical RP HPCL profiles of reaction mixture of synthesis of oligonucleotides; Figure S2: Analytical RP HPCL profiles of purified oligonucleotides; Figure S3: Results of ESI mass spectrometry; Figure S4: Correlogram, intensity and number graphs of DLS experiment; Table S1: Melting temperatures.
