# Supplementary material for: Influence of Combinations of Lipophilic and Phosphate Backbone Modifications on Cellular Uptake of Modified Oligonucleotides
Source: Molecules. 2024 Jan 17;29(2):452. doi: 10.3390/molecules29020452 (PMC10818405; doi:10.3390/molecules29020452)
Supplement: Supplementary file 1 [file molecules-29-00452-s001.zip › molecules-2790845-supplementary.pdf]

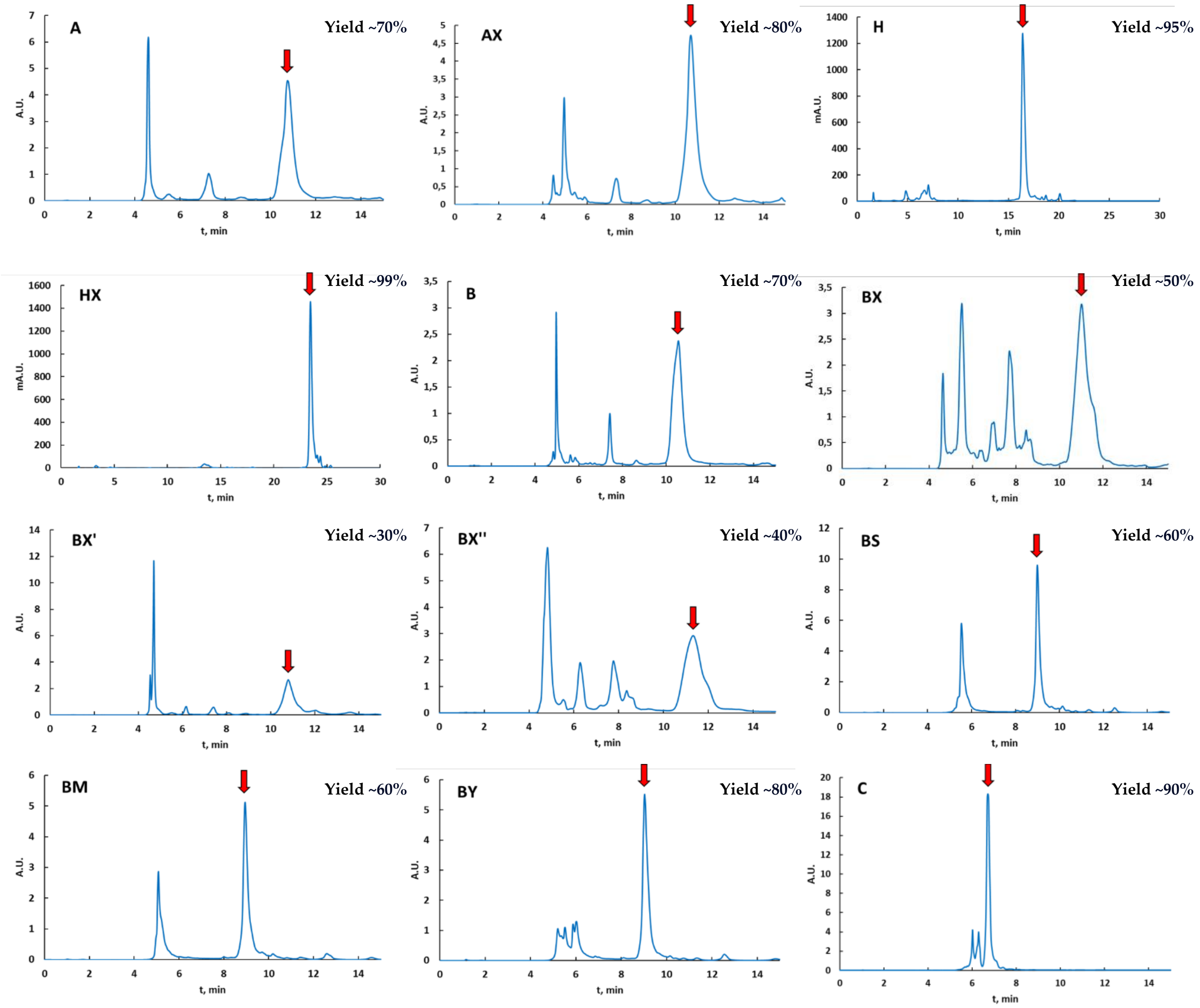

Figure S1. Analytical RP HPCL profiles of reaction mixture of synthesis of corresponding modified oligonucleotides. Red arrow shows peak that was collected and identified as desired product. Oligonucleotides H, HX containing cholesterol moieties were analyzed using preparative 4.6×150 mm C4 column in the gradient from 0 to 90% of acetonitrile in 0.02 M tetraethylammonium acetate (TEAAc) in 30 minutes. The other oligonucleotides were analyzed using analytical 2×75 mm C18 column in the gradient from 0 to 90% (or from 0 to 50% for oligonucleotide C) of acetonitrile in 0.02 M tetraethylammonium acetate (TEAAc) in 15 minutes. Yields were calculated as the ratio of the peak area of the desired product to the total absorption at the wavelength 260 nm.

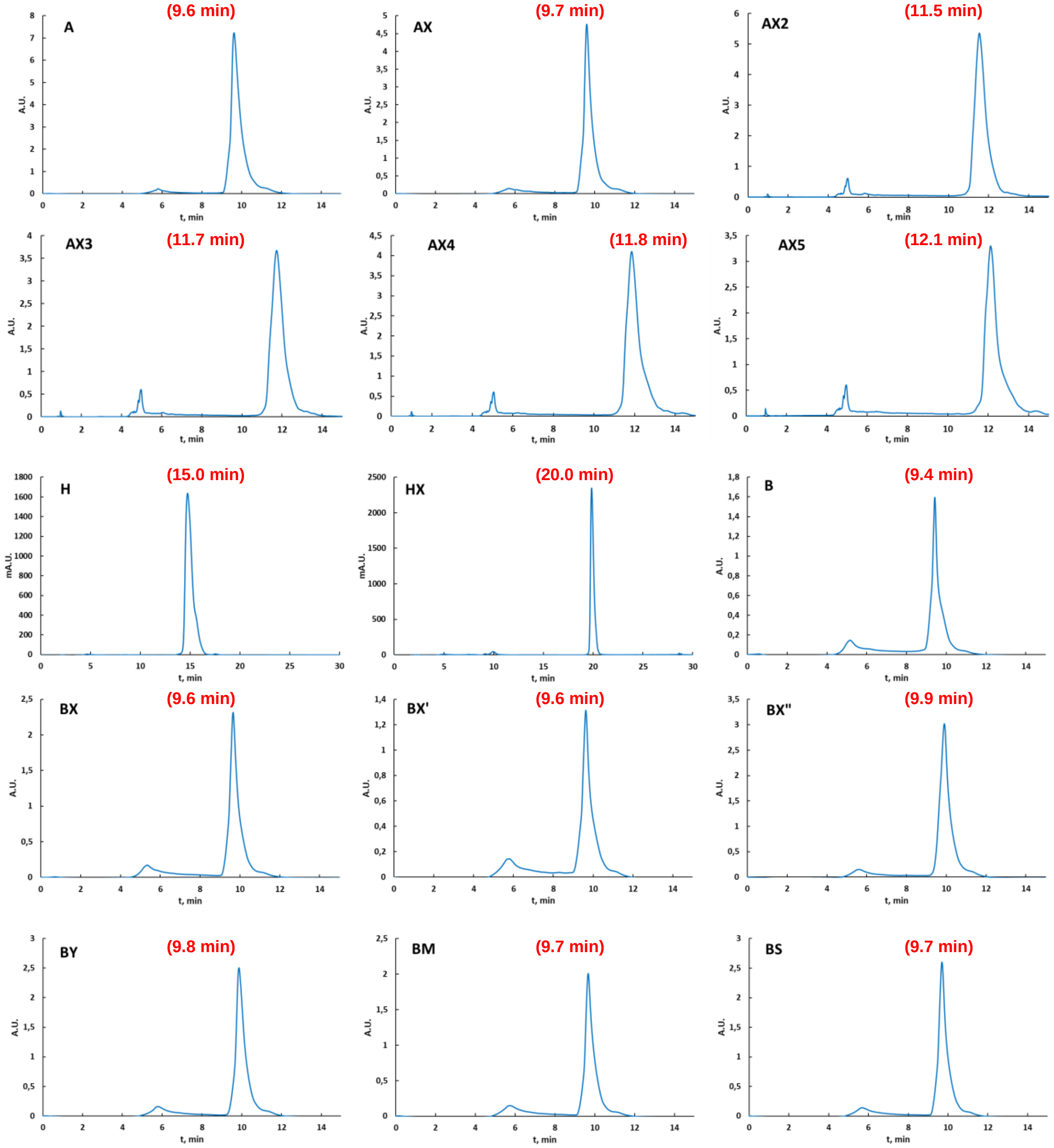

Figure S2. Analytical RP HPCL profiles of purified oligonucleotides. Oligonucleotides H, HX containing cholesterol moieties were analyzed using preparative 4.6×150 mm C4 column in the gradient from 0 to 90% of acetonitrile in 0.02 M tetraethylammonium acetate (TEAAc) in 30 minutes. The other oligonucleotides were analyzed using analytical 2×75 mm C18 column in the gradient from 0 to 90% of acetonitrile in 0.02 M tetraethylammonium acetate (TEAAc) in 15 minutes. Some impurities with a retention time of 5-6 minutes indicates the column artifacts of non-nucleotide nature.

SUPPLEMENTARY MATERIAL S3

A 5′-[FAM]CTGACTATGAAGTAT*T-3′

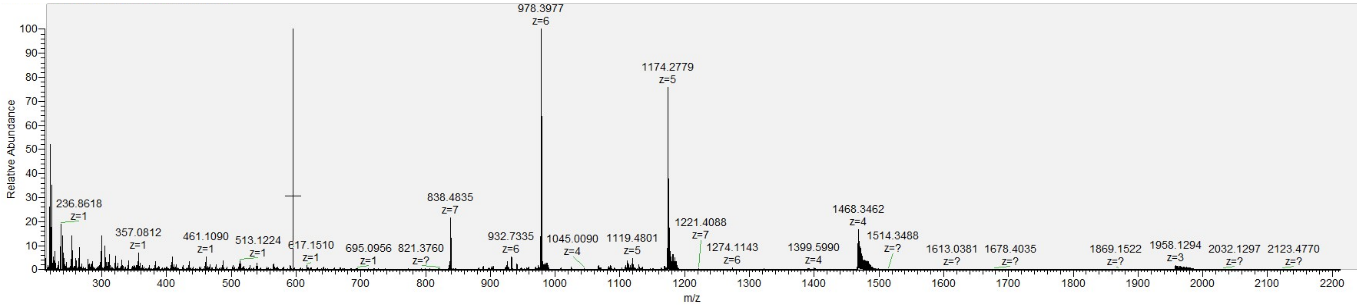

AX 5′-[FAM]^{x}C^{x}TGACTATGAAGTAT*T-3′

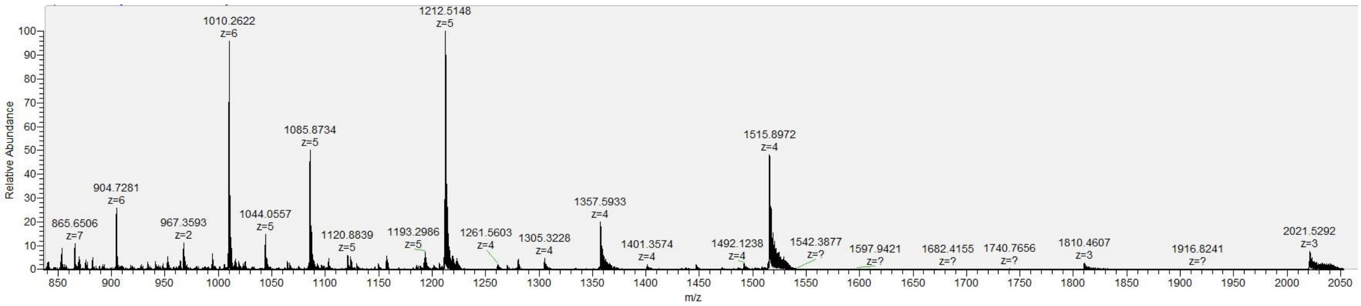

AX2 5′-[FAM]^{x}C^{x}T^{x}G^{x}ACTATGAAGTAT*T-3′

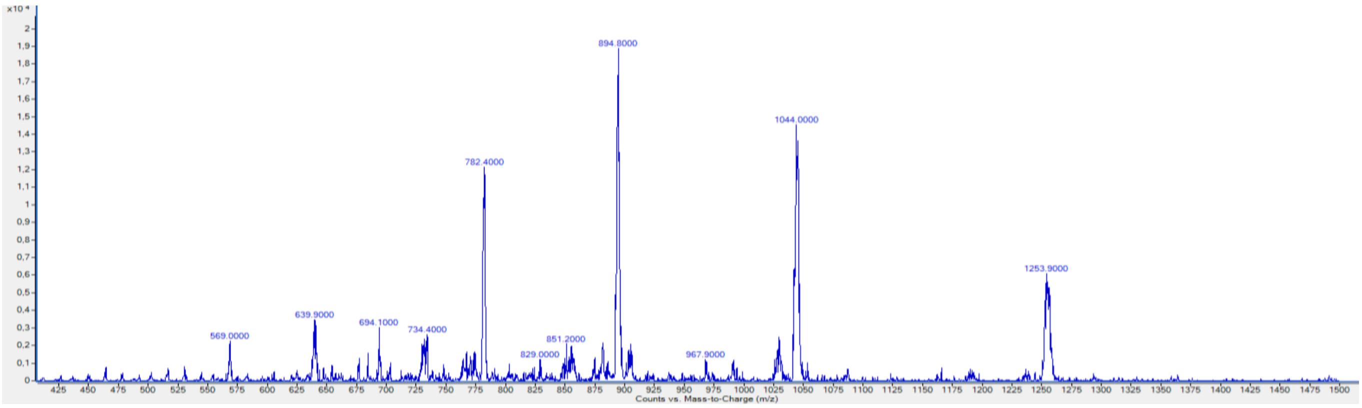

AX3 5′-[FAM]^{x}C^{x}T^{x}G^{x}A^{x}C^{x}TATGAAGTAT*T-3′

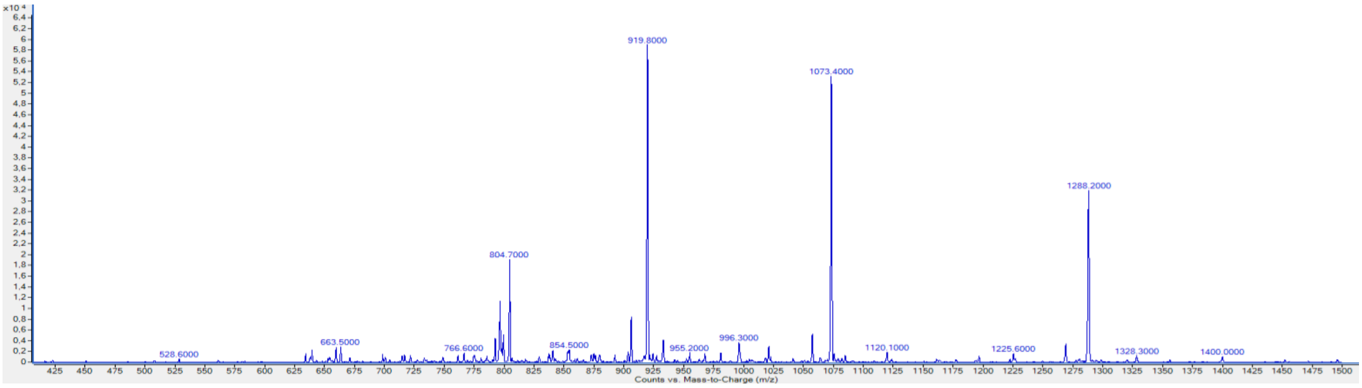

SUPPLEMENTARY MATERIAL S3 (Continued)

AX4 5′-[FAM]^{x}C^{x}T^{x}G^{x}A^{x}C^{x}T^{x}A^{x}TGAAGTAT*T-3′

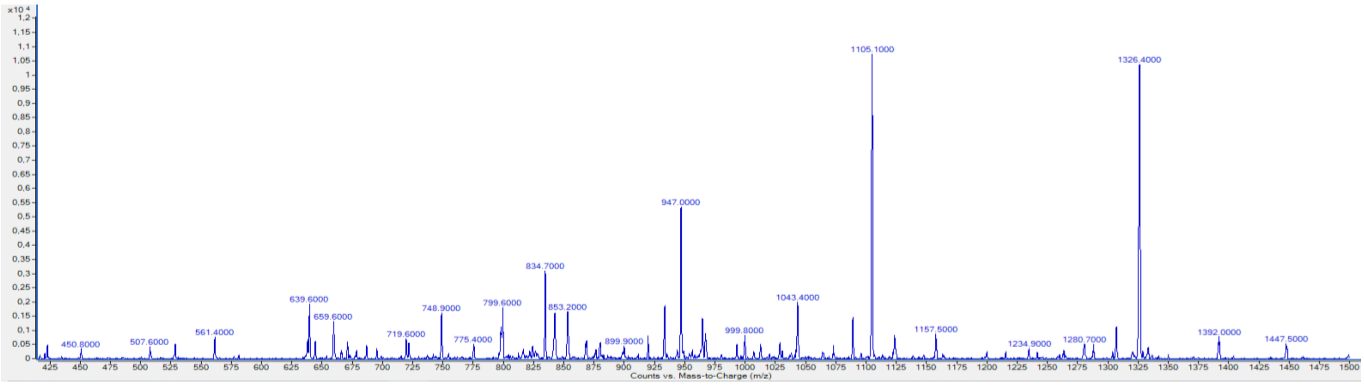

AX5 5′-[FAM]^{x}C^{x}T^{x}G^{x}A^{x}C^{x}T^{x}A^{x}T^{x}G^{x}AAGTAT*T-3′

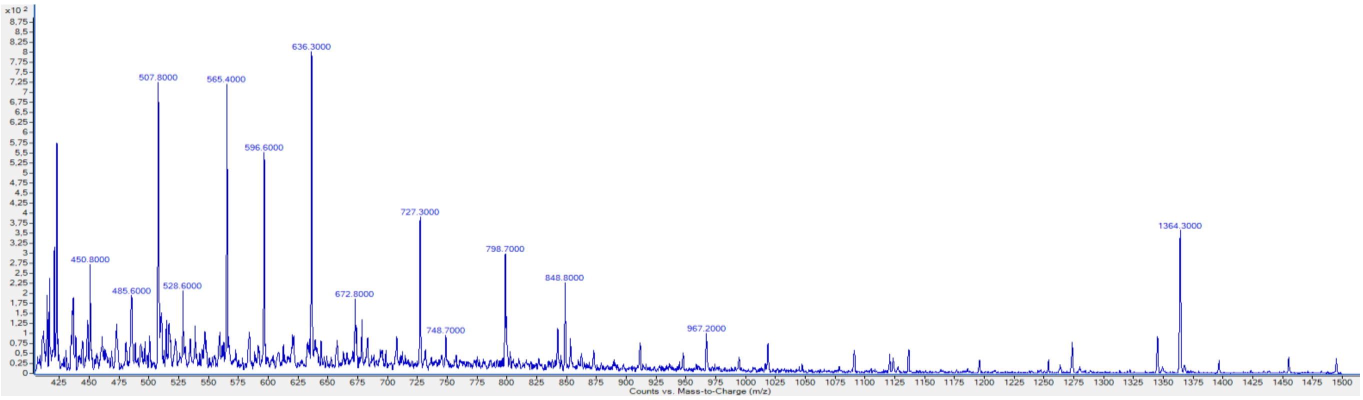

H 5′-[FAM]CTGACTATGAAGTATT[Chol]-3′

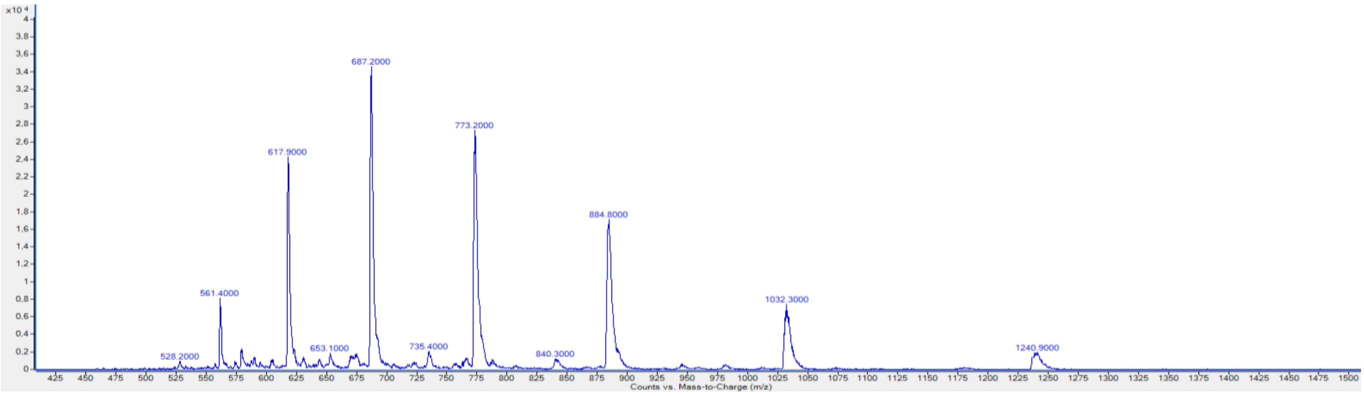

HX 5′-[FAM]^{x}C^{x}TGACTATGAAGTATT[Chol]-3′

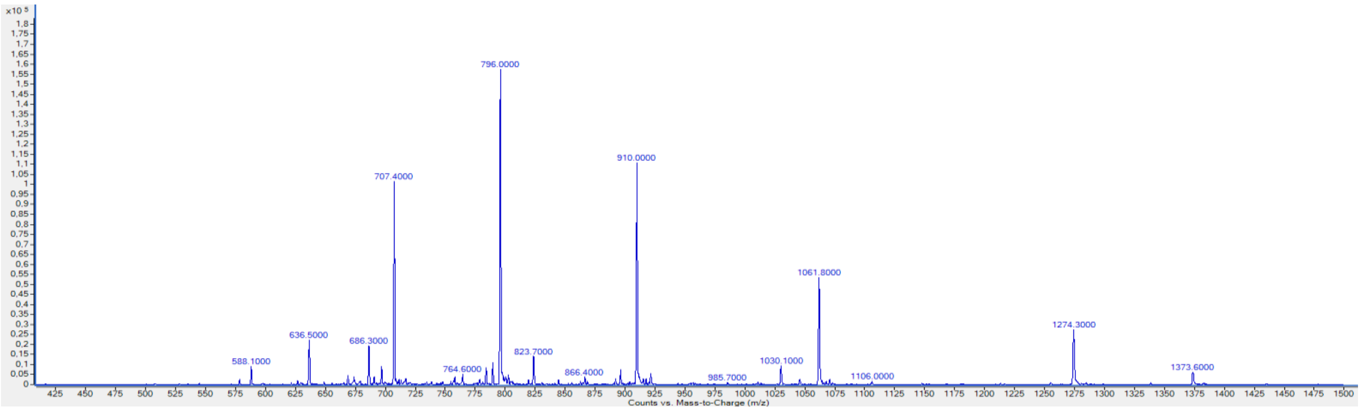

SUPPLEMENTARY MATERIAL S3 (Continued)

B 5′-[FAM]AGTCTCGACTTGCTAT*T-3′

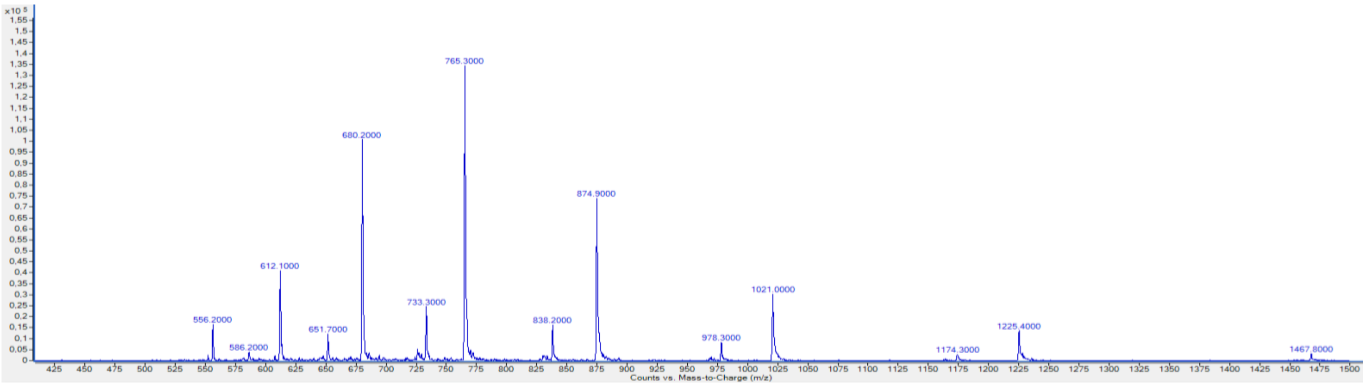

BX 5′-[FAM]xAxGTCTCGACTTGCTAT*T-3′

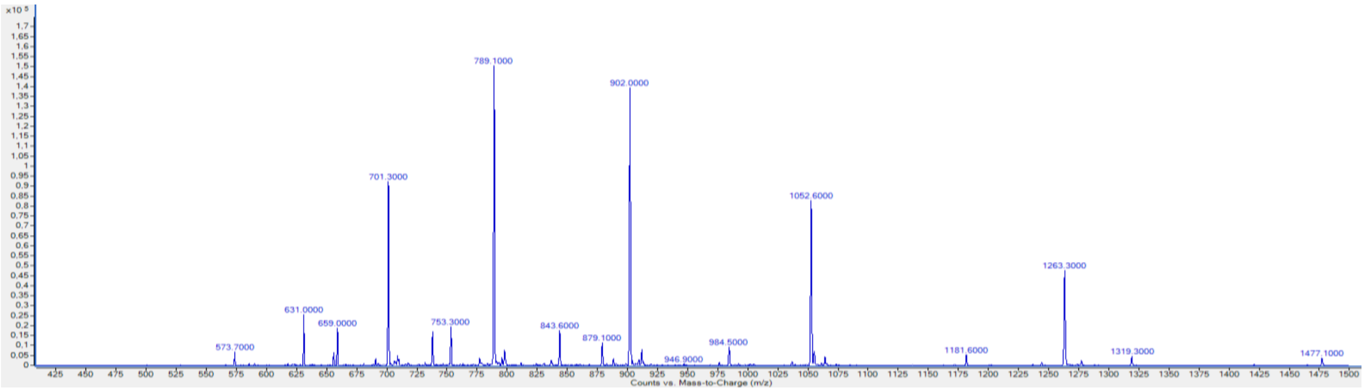

BX′ 5′-[FAM]AGTCTCGxAxCTTGCTAT*T-3′

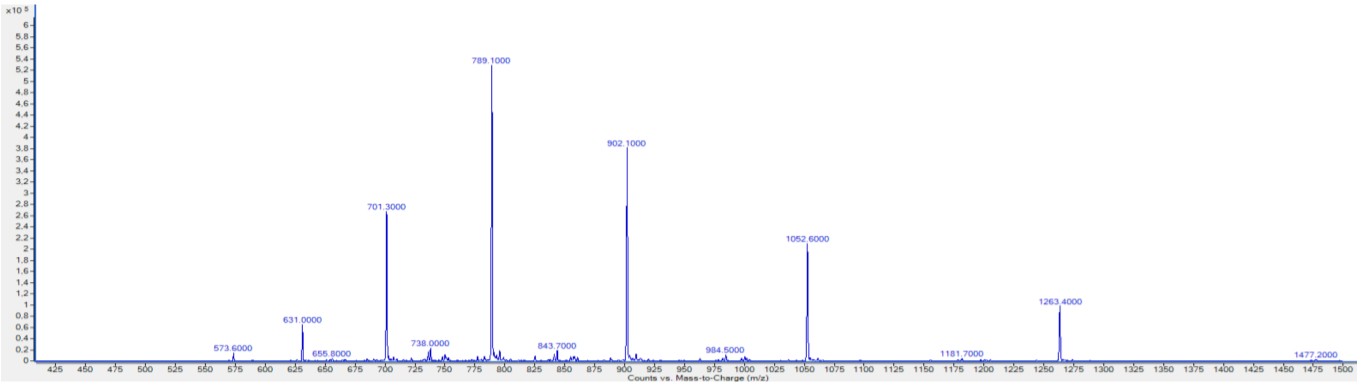

BX′′ 5′-[FAM]AGTCTCGACTTGCTxAxT*T-3′

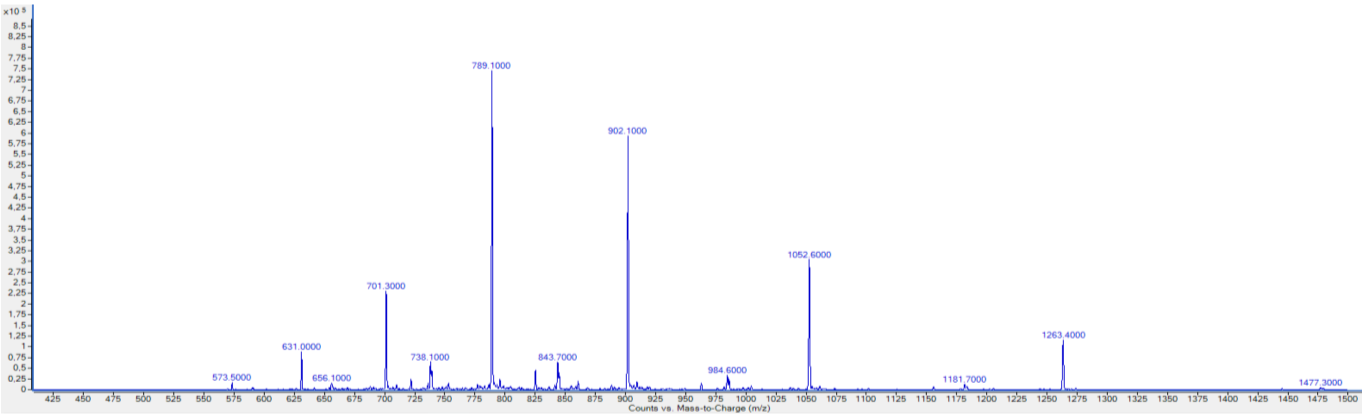

SUPPLEMENTARY MATERIAL S3 (Continued)

BS 5′-[FAM]^{S}A^{S}GTCTCGACTTGCTAT*T-3′

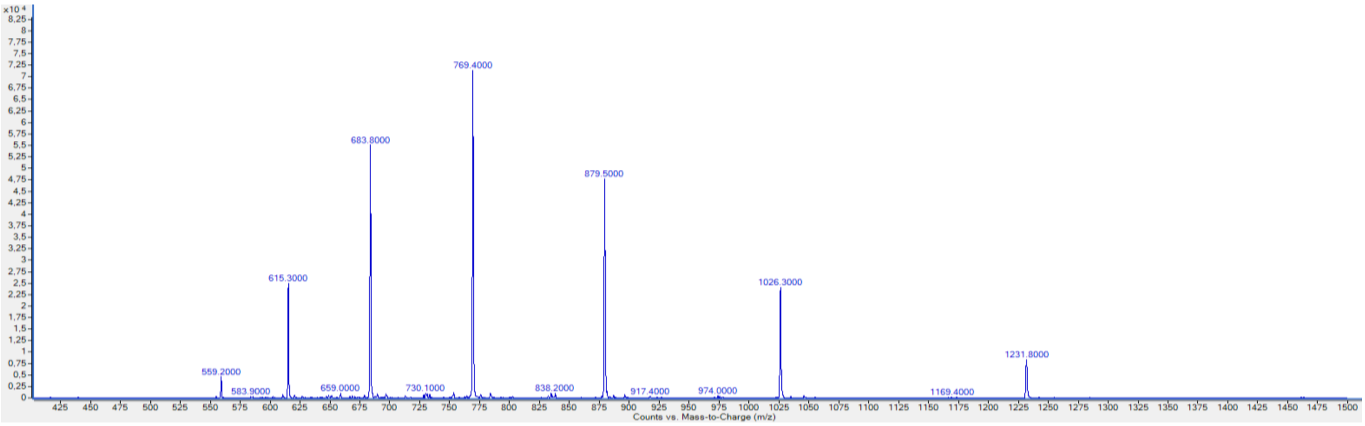

BY 5′-[FAM]^{Y}A^{Y}GTCTCGACTTGCTAT*T-3′

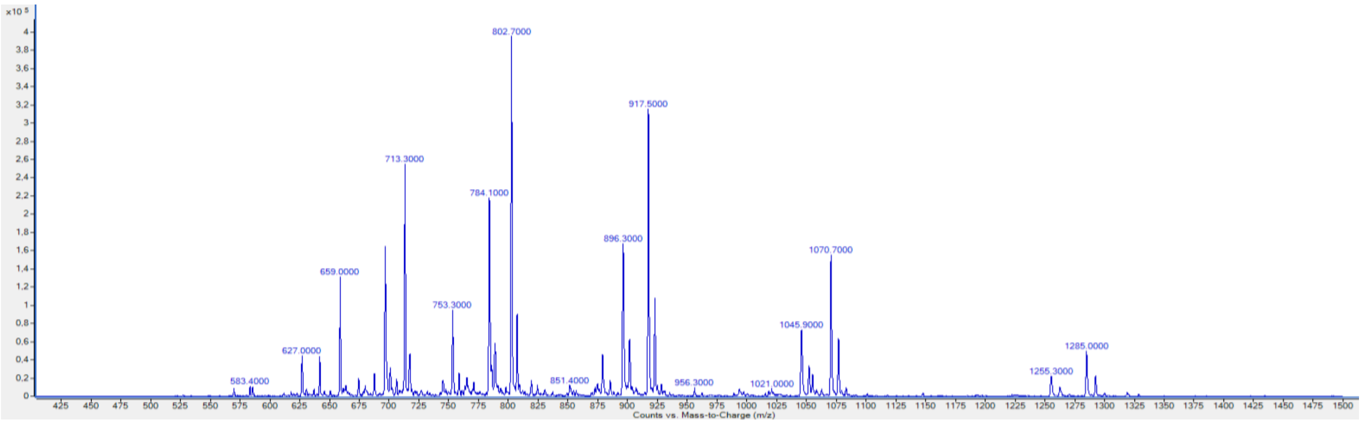

BM 5′-[FAM]^{M}A^{M}GTCTCGACTTGCTAT*T-3′

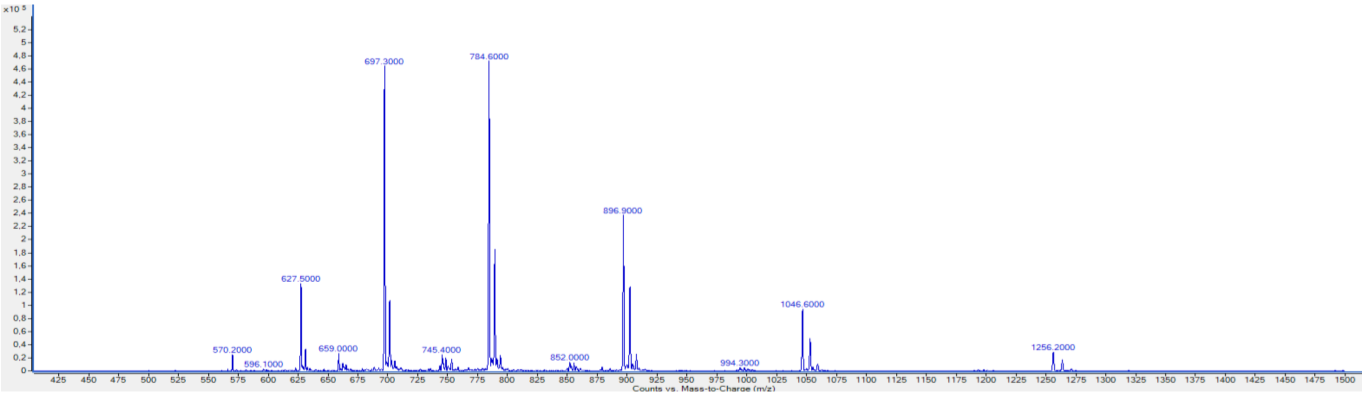

Figure S3. Undeconvoluted results of ESI mass spectrometry in negative ion registration mode of corresponding modified oligonucleotides.

SUPPLEMENTARY MATERIAL S4

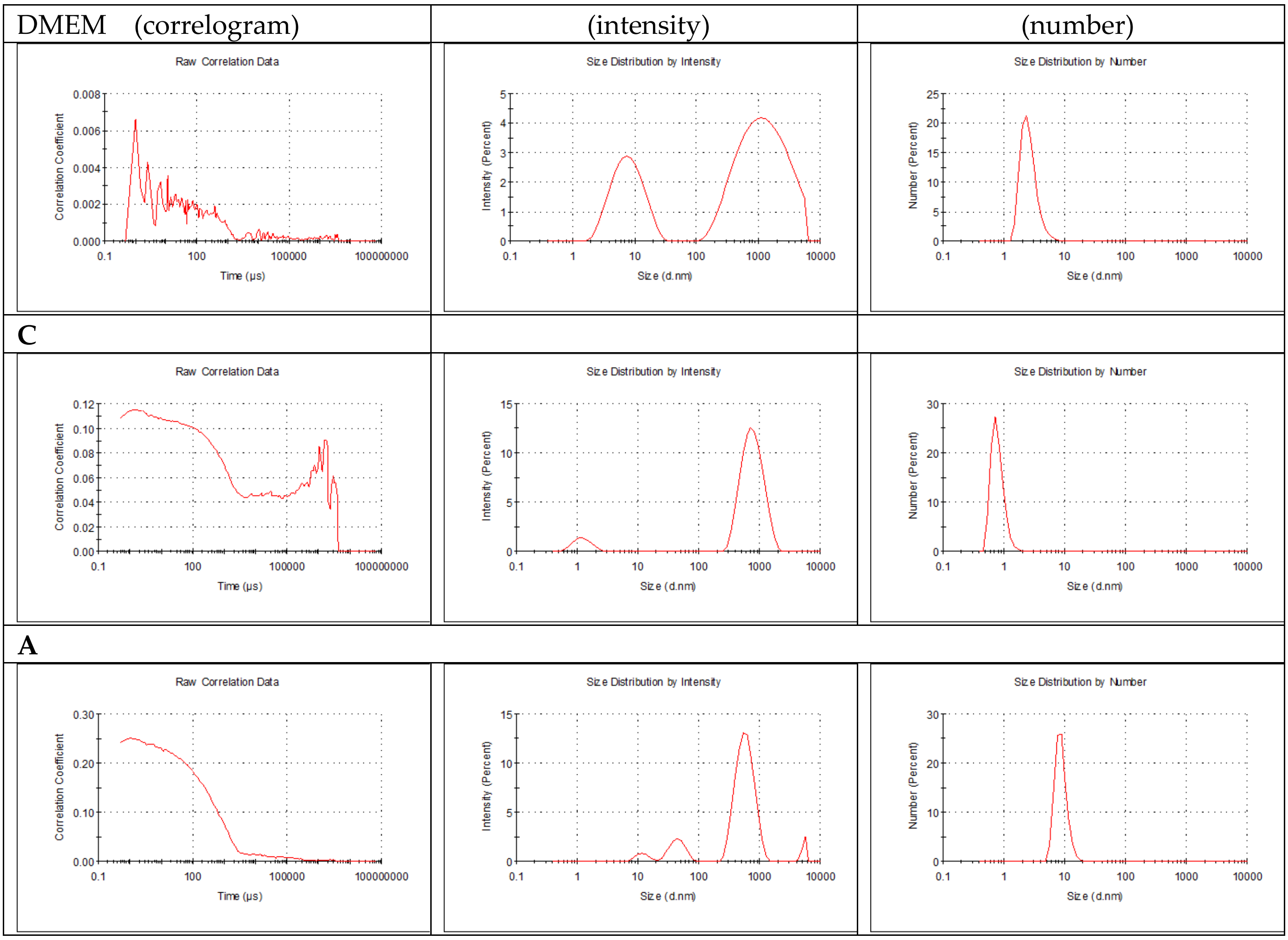

Figure S4. Correlogram, intensity and number graphs of DLS experiment for the controls (DMEM medium, unmodified oligonucleotide C) and oligonucleotide A. Measurements conditions: DMEM medium, 10 measurements in 2 h, 5µM concentration of oligonucleotide.

SUPPLEMENTARY MATERIAL S5

Table S1. Melting temperatures for duplexes of studied oligonucleotides with complementary unmodified oligonucleotides M, 5'-AAT ACT TCA TAG TCA G-3' and N, 5'- AAT AGC AAG TCG AGA CT -3'; F – fluoresceine labeled oligonucleotide with the sequence of 5'-CTG ACT ATG AAG TAT T-3'. Thermal denaturation experiments were carried out using a UV detector on a UV-1800 UV spectrophotometer (Shimadzu, Japan) equipped with a Peltier block. Equimolar amounts (4 μM, 50 μL of each strand) of complementary oligonucleotides were used. Melting curves were recorded at 260 nm within a temperature range from 25°C to 85°C with a heating/cooling rate of 0.2°C/min, in a x1 PBS buffer.

Code	T_m, °C	ΔT_m, °C
F/M	50.3	-
A/M	49.8	-0.5
AX/M	46.1	-4.2
AX2/M	43.5	-6.8
AX3/M	39.4	-10.9
AX4/M	38.5	-11.8
AX5/M	38.6	-11.7
H/M	53.0	+2.7
HX/M	50.9	+0.6
C/N	60.5	-
B/N	61.2	+0.7
BX/N	58.5	-2.0
BX′/N	59.5	-1.0
BX″/N	59.3	-1.2
BS/N	60.7	+0.2
BM/N	60.1	-0.4
BY/N	59.1	-1.4
